# Status, quality and specific needs of Ebola virus diagnostic capacity and capability in laboratories of the two European preparedness laboratory networks EMERGE and EVD-LabNet

**DOI:** 10.2807/1560-7917.ES.2018.23.19.17-00404

**Published:** 2018-05-10

**Authors:** Chantal B Reusken, Ramona Mögling, Pieter W Smit, Roland Grunow, Giuseppe Ippolito, Antonino Di Caro, Marion Koopmans

**Affiliations:** 1 Department of Viroscience, World Health Organization Collaborating Centre for Arbovirus and Viral Haemorrhagic Fever Reference and Research, Erasmus University Medical Centre, Rotterdam, the Netherlands; 2 Robert Koch Institute, Berlin, Germany; 3 National Institute for Infectious Diseases (INMI) Lazzaro Spallanzani, Rome, Italy

**Keywords:** ebola virus, diagnostics, preparedness, emergency response, capacity, biosafety

## Abstract

From December 2013 to March 2016, West Africa experienced the largest Ebola virus (EBOV) outbreak to date, leading to a European-wide activation of laboratory preparedness and response. At the end of the outbreak, laboratories associated with the two European preparedness networks of expert laboratories EMERGE JA and EVD-LabNet were invited to participate in an assessment of the response of European laboratories to the EBOV outbreak, to identify learning points and training needs to strengthen future outbreak responses. Response aspects assessed included diagnostics, biorisk management and quality assurance. The overall coverage of EBOV diagnostics in the European Union/European Economic Area (EU/EEA) was found to be adequate although some points for quality improvement were identified. These included the need for relevant International Organization for Standardization (ISO) accreditation, the provision of EBOV external quality assessments (EQA) in periods where there is no emergency, facilitating access to controls and knowledge, biorisk management without compromising biosafety and a rapid public health response, and the need for both sustained and contingency funding for preparedness and response activities.

## Background

From December 2013 onward, the world experienced the largest Ebola virus (EBOV) outbreak to date, with more than 28,000 cases including more than 11,000 deaths mostly in Guinea, Liberia and Sierra Leone [1]. The outbreak with EBOV strain Zaire in West Africa was declared a public health emergency of international concern (PHEIC) by the World Health Organization (WHO) between 8 August 2014 and 29 March 2016 [2,3]. Upon declaration of the PHEIC, the European Centre for Disease Prevention and Control (ECDC) forecast that despite low probability of imported cases, a substantial number of people would need to be investigated to rule out EBOV infection in the European Union (EU) and the European Economic Area (EEA) during the outbreak [4]. A modelling study listed four European countries (Belgium, France, Germany and the United Kingdom (UK)) in the 16 countries most at risk for importation of EBOV, while three additional European countries (Italy, the Netherlands and Spain) were modelled to be at risk for transiting EBOV-infected travellers [5]. A risk for local transmission should also be considered in case of repatriation of patients or accidentally exposed persons (e.g. healthcare workers and/or laboratory personnel) to European countries [6,7].

Two laboratory networks were actively involved in provision of EBOV diagnostic support: (i) the EU-funded joint-action initiative QUANDHIP (continued as EMERGE JA) that focused on high-consequence cross-border threats (highly pathogenic bacteria and risk group 4 (RG4) viruses) and was activated by the EU Health Security Committee (HSC) in August 2014, and (ii) the ECDC-funded European Network for Imported Viral Diseases (ENIVD, now named EVD-LabNet [8-10]). In September 2014, an assessment through the ENIVD network showed that 31 laboratories, including eight biosafety level four (BSL4) facilities, were able to handle EBOV diagnostic requests, and it was concluded that Europe had sufficient laboratory capacity to detect imported EBOV cases [11]. After the end of the PHEIC, EMERGE JA conducted an inventory of the demands on EBOV diagnostic capacity and capability during the outbreak in European laboratories associated with its network and EVD-LabNet. This was done to gain insight into problems encountered, protocols used and lessons learned and to identify needs for training and other improvements to strengthen the European laboratory response to future outbreaks.

## Methods

Laboratories associated with the two European preparedness networks of expert laboratories EMERGE JA [10] and EVD-LabNet [8] were asked to complete an online questionnaire (available from authors upon request) to assess the European laboratory response to the EBOV outbreak in West-Africa. 

These included 38 laboratories in 23 EU/EEA countries and one non-EU/EEA country associated with EMERGE JA as well as 58 laboratories in 27 EU/EEA countries, three EU candidate countries and one non-EU/EEA country associated with EVD-LabNet. Among these, 23 laboratories were members of both networks and were asked to fill in the questionnaire only once. 

The questionnaire was designed to address essential aspects of response targeting (i) EBOV diagnostics, (ii) biorisk management and (iii) quality assurance, while identifying (iv) challenges and needs. The questionnaire was sent on 13 July 2016 to all member institutes of both networks and closed on 20 September 2016. The involved laboratories where either national and international reference centres for EBOV diagnostics or regional laboratories possibly involved in the screening of suspects.

## Results

### Survey response

**Figure 1:**
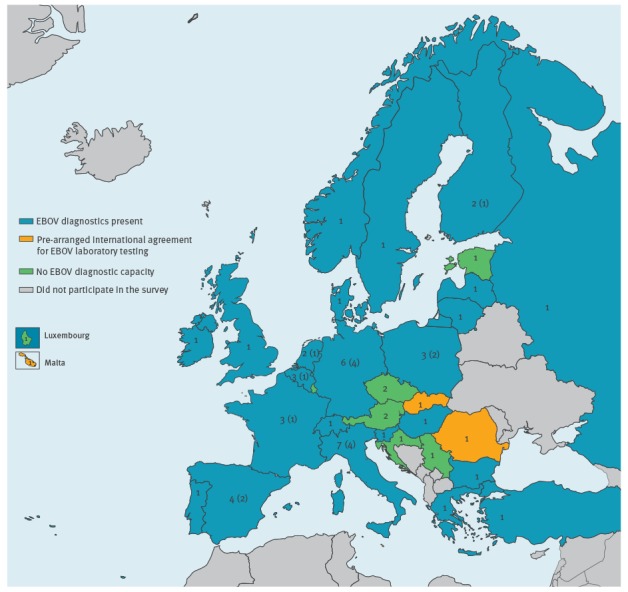
Availability of Ebola virus diagnostics in EU/EEA, EU candidate and non-EU/EEA countries, September 2016 (n = 56)

EBOV: Ebola virus; EEA: European Economic Area; EU: European Union.

Of the 69 invited laboratories, representatives from 56 laboratories in 28 EU/EEA countries, two EU candidate countries, and two non-EU/EEA European countries completed the questionnaire (Figure 1). All 23 laboratories that were members of both EMERGE JA and EVD-LabNet responded. Twenty-eight of 35 members of EVD-LabNet alone and five of 14 laboratories associated with EMERGE JA alone responded. Of the eight BSL4 laboratories that were operational to conduct EBOV diagnostics during the PHEIC (Budapest, Hamburg, Lyon, Marburg, Porton Down, Rome and Stockholm in EU/EEA countries, and Spiez in a non-EU/EEA country), all but one participated in the survey.

The total number of laboratories that participated in the questionnaire is indicated per country. For countries with EBOV diagnostics and more than one laboratory participating in the survey, the number of laboratories with EBOV diagnostics is given in brackets.

### Availability of Ebola virus diagnostics and level of preparedness

Thirty-one laboratories in 20 EU/EEA countries, one EU candidate country and two non-EU/EEA countries performed EBOV diagnostics (Figure 1). Laboratories in two of eight countries without EBOV diagnostics indicated that they had a pre-agreement with a laboratory in another country to perform EBOV diagnostics (Figure 1). Of the 31 laboratories with EBOV diagnostics, 15 laboratories in 12 countries indicated to have implemented EBOV diagnostics for the first time during the 2014-16 outbreak in West Africa, while 13 countries already had national laboratories with EBOV diagnostics before that outbreak. In the following, we concentrate specifically on the survey responses from those 31 EBOV diagnostic laboratories only. When fewer than 31 participants answered a question this is explicitly indicated.

Twenty-six laboratories in 21 countries enrolled and/or trained additional staff in response to the outbreak. Twenty-three laboratories in 19 countries prepared new or updated existing standard operating procedures (SOPs) at the beginning of the PHEIC, while 19 laboratories in 17 countries indicated to have revised SOPs based on lessons learned during the PHEIC. Of the seven BSL4 laboratories, four laboratories indicated to have implemented new SOPs. These SOPs were all related to introduction of new commercial tests as back-up or replacement of existing tests. An inventory of the type of SOPs that were adapted during the outbreak is given in Table 1. Only 14 laboratories in 11 countries indicated to have received additional funding to respond to the EBOV outbreak.

**Table 1:** Overview of standard operating procedures prepared or updated at the start of and during the Ebola virus PHEIC by European Ebola virus diagnostic laboratories (n = 31 laboratories, n = 23 countries)

SOP topic	Start^a^	During^a^
Differential diagnosis, parallel testing other pathogens	2	1
Adaptation malaria diagnostics to BSL3 setting	1	0
EBOV testing upon implementation of new test, adapted test, additional targets	14	5
Workflow from biosafety perspective including sample taking, waste disposal, handling before and after inactivation, before and after BSL3	13	6
Usage of personal protective equipment	9	9
Shipment from peripheral laboratories to reference laboratory	3	0
Adaptation of case definition	1	0
Introduction of bedside blood inactivation	0	1
Revision of SOPs and training courses for personnel	0	1
Change inactivation procedure	0	4

BSL: biosafety level; PHEIC: public health emergency of international concern; SOP: standard operating procedure.

^a^ Number of laboratories indicating preparation or revision of the indicated SOP at the start or during the EBOV outbreak.

### Molecular, serological and differential diagnostics

All EBOV diagnostic laboratories performed molecular EBOV assays and had access to a positive control, including virus controls provided in commercial PCR kits. Overall, the laboratories indicated the use of a total of eight different commercial and 11 published in-house tests. For five in-house tests, no background information was given (Table 2).

**Table 2:** Overview of in-house and commercial Ebola virus molecular tests used by European Ebola virus diagnostic laboratories (n = 31 laboratories, n = 23 countries)

In-house	Total number of laboratories (countries)	Commercial	Total number of laboratories (countries)
Panning et al., 2007 [37]	5 (3)	Altona	22 (16)
Gibb et al., 2001 [38]	4 (4)	Cepheid	2 (2)
Ogawa et al., 2011 [39]	5 (5)	Roche	2 (2)
Sanchez et al., 1999 [40]	3 (3)	Biofire filmarray	2 (2)
Trombley et al., 2010 [41]	4 (4)	Genesig	2 (2)
Huang et al., 2012 [42]	2 (2)	Amplisens	1 (1)
Dedkov et al., 2016 [43]	1 (1)	Bioline sensifast	1 (1)
Jaaskelainen et al., 2015 [44]	1 (1)	Sacace	1 (1)
Weidmann et al., 2004 [45]	1 (1)		
De la Vega et al., 2015 [46]	1 (1)		
CDC, 2015 [47]	1 (1)		
In-house not specified	5 (3)		

CDC: Centers for Disease Control and Prevention.

Assessment of the level of molecular test result confirmation revealed that, of 23 laboratories that answered this question, 14 laboratories in 12 countries confirmed both positive and negative test results either through a second PCR test targeting another part of the EBOV genome or by sending the sample to a reference laboratory. Six laboratories in five countries only sought confirmation of positive test results while three in three countries, including one BSL4 laboratory, never confirmed a test result. At the country level this translated to one country without any EBOV test confirmation and four countries with only confirmation of positive results. Eighteen of 25 laboratories that answered this question indicated that they had the possibility to refer samples to one or more BSL4 laboratories. The indicated reference BSL4 laboratories were Hamburg (n = 8), Marburg (n = 1), Porton Down (n = 2), Rome (n = 2) and Stockholm (n = 3) or ‘any laboratory within EMERGE JA’ (n = 2).

In-house serological assays were performed by nine laboratories in eight countries, which included five BSL4 laboratories. For seven of these in-house tests, a positive control was available. No further details about serology were asked as the role of serology in EBOV diagnostics is limited. 

**Figure 2:**
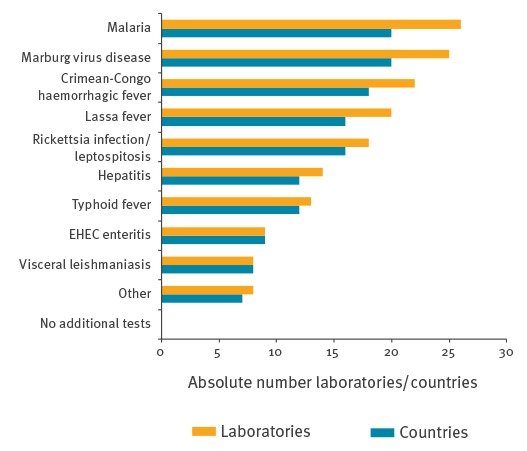
Available differential diagnostics in European Ebola virus diagnostic laboratories (n = 31) and number of countries they represent (n = 23)

All laboratories indicated that they performed tests for additional pathogens that, based on expert opinion, could be part of the differential diagnosis of suspected EBOV patients (Figure 2). The BSL4 laboratories all provided differential diagnostics for three other relevant RG4 pathogens (Crimean-Congo haemorrhagic fever virus, Lassa virus and Marburg virus) as did nine BSL3 laboratories in eight countries.

EHEC: Enterohaemorrhagic *Escherichia coli*.

‘Other’ included laboratories that indicated tests for dengue virus, chikungunya virus, varicella-zoster virus and herpes simplex virus.

To gain insight in the diagnostic burden, participants were asked how many samples were tested for EBOV in their laboratory in the period from March 2014 to September 2015. This question addressed the actual diagnostic burden in laboratories in Europe regardless of the origin of the diagnostic samples, i.e. whether from within or outside Europe. Four laboratories did not provide data and four laboratories in four countries indicated they had not had any diagnostic requests for EBOV in this period. The remaining 23 laboratories in 20 countries tested a total of 3,342 samples of which 2,868 (86%) were performed in the seven institutes with a BSL4 facility. Both sample counts consisted of responses defining precise numbers and responses giving a rounded estimate. A total of 253 samples of the submitted 3,342 were positive for EBOV RNA in six BSL4 and two BSL3 facilities in eight countries. The median time from arrival of the sample at the 23 laboratories to the outcome of the EBOV test result was 7 hours (range: 3.5–48 hours).

Fifteen laboratories in 11 countries, including all but one participating BSL4 laboratories, provided support to other (inter)national laboratories in the form of shared protocols, assays and/or positive control material. When asked about the quality of collaboration among EU bodies and organisations in terms of sharing knowledge, e.g. SOPs, and materials for EBOV diagnostics, the 30 responding laboratories gave a favourable evaluation on a Likert-scale from 0 = poor to 6 = excellent: the median for both networks, excluding the BSL4 laboratories, was 5 (range: 1–6), while the seven BSL4 laboratories rated a 4 (range: 2–5).

### Biorisk management

To gain insight in the operational BSL in the European laboratories that conducted first-line and/or reference EBOV diagnostics and in the modifications applied to address clinical requests in addition to EBOV exclusion testing, participants indicated the BSL used for EBOV diagnostics. Five laboratories in five countries conducted EBOV diagnostics at BSL4, whereas 24 laboratories in 18 countries performed EBOV diagnostics at BSL3, including two laboratories with BSL4 facilities. One of two laboratories performing EBOV diagnostics at BSL2 indicated to do so upon bedside inactivation of samples from suspected EBOV patients. At the country level, this corresponded to four countries with EBOV diagnostics only at BSL4, one country with EBOV diagnostics at all three biosafety levels (BSL4, 3 and 2) depending on the laboratory, and one country with EBOV diagnostics only at BSL2. Seventeen countries had EBOV diagnostics only at BSL3. Twenty-one of 24 laboratories performing EBOV diagnostics at BSL3 indicated to take extra precautionary measures in addition to regular BSL3 protocols. These measures included extra personal protective equipment (PPE) in addition to normal BSL3 PPE for 14 laboratories. The use of goggles/face mask, coverall/head coverage, respirator, waterproof apron/long sleeves and boot covers, and the obligation to shower-out were indicated as adaptations. Fifteen laboratories had implemented additional sample inactivation into the BSL3 routine, either by heat inactivation or addition of 96% ethanol to the lysis buffer [12].

Seven laboratories in seven countries among the 30 laboratories that answered this question also performed clinical biochemistry and haematology on samples from suspected or confirmed EBOV cases and indicated to do so at BSL2 (n = 1), BSL3 (n = 5) or BSL4 (n = 1). The laboratory that performed clinical biochemistry and haematology at BSL2 conducted EBOV diagnostics at BSL3.

Disinfection protocols to re-use equipment in case an EBOV case was confirmed were in place in 24 laboratories of 28 laboratories that answered this question. Ten laboratories in eight countries indicated that the laboratory had to be closed for the disinfection process and that this affected routine diagnostics.

Of the eight laboratories in eight countries that had received EBOV-positive diagnostic samples, four (two BSL4 and two BSL3 laboratories) indicated that they had implemented a special health surveillance protocol (e.g. temperature monitoring for 21 days) for laboratory employees who handled EBOV-positive specimens, while four laboratories (three BSL4 and one BSL3) had not.

### Quality assurance

To gain insight in the level of quality control at the EBOV diagnostic laboratories, laboratories were asked to specify their level(s) of laboratory accreditation. Twenty-one laboratories worked under a relevant International Organization for Standardization (ISO) accreditation scheme (ISO 15189 [13], ISO 17025 [14], ISO 9001 [15]), of which 12 under ISO 15189 that is recommended for medical diagnostic laboratories [13] while 10 laboratories had no ISO accreditation at all. It is noteworthy that only four of the seven BSL4 laboratories performed diagnostics under an ISO accreditation of which two were under ISO-15189. Analysis at the country level showed 10 countries with one and one country with two EBOV laboratories with ISO 15189 accreditation. Five countries had no national EBOV diagnostic laboratory with an ISO accreditation of which one indicated to have a national accreditation.

Another aspect of quality assurance is the participation of laboratories in external quality assessment (EQA) exercises. During the EBOV outbreak, two open international EQAs for testing of EBOV RNA were organised by WHO and ENIVD [16]. Nine of 30 responding laboratories participated in the WHO EBOV EQA, while 26 participated in the ENIVD EQA [16]. Five BSL4 laboratories indicated to have participated in the closed QUANDHIP EBOV EQAs between 2012 and 2015. Two laboratories with EBOV diagnostics indicated not to have participated in any of the EBOV EQAs. At the country level, this translated to two countries with an EBOV diagnostic laboratory without external quality assessment.

Finally, quality can be assured by using official diagnostic algorithms. Nine laboratories performed EBOV diagnostics based on the ECDC diagnostic algorithm. Four laboratories followed the WHO algorithm and five followed national guidelines. There were three laboratories that indicated to have adopted both ECDC and WHO guidelines. Ten laboratories did not answer this question.

### Challenges and needs

**Figure 3:**
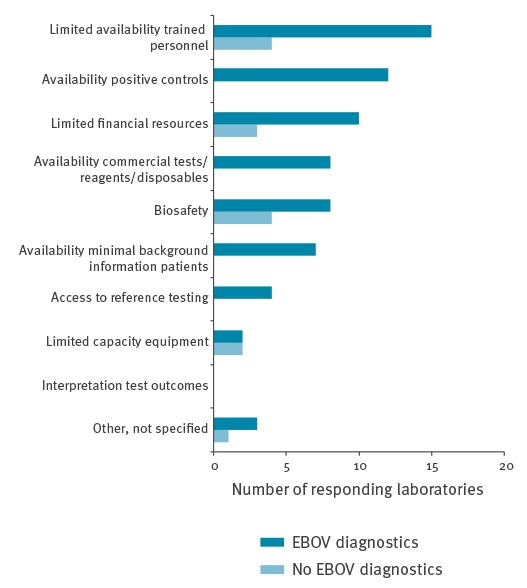
Challenges for laboratories to respond to the Ebola virus outbreak in West Africa (n = 37; 29 performing and eight not performing Ebola virus diagnostics)

Laboratories were asked to identify the main challenges they faced in connection with response to the EBOV outbreak in West Africa (Figure 3). The question was addressed by 29 EBOV diagnostic laboratories and eight non-EBOV diagnostic laboratories. The main challenges were the availability of trained personnel and positive control materials. The BSL4 laboratories indicated availability of funds and trained personnel as main issues. For laboratories that did not perform EBOV diagnostics during the outbreak, biosafety was among the main concerns.

When asked to identify training needs, 12 of 30 responding laboratories indicated that the protocols and response they had set up before the outbreak worked well and no further training was necessary. Seven laboratories suggested that a workshop on differential EBOV diagnosis both for molecular and serological tests would be useful. Five laboratories asked for training courses in biosafety and four laboratories asked for PPE procedures. Further individual requests included courses on: laboratory sample handling, decontamination of rooms and cars, training of primary responders including bedside routine analyses, and advice on the best commercials assay systems.

Finally, laboratories were asked to give suggestions that could help improve preparedness and response in case of a future outbreak. Three main topics emerged: media communication, speed of the outbreak response and staff training. One laboratory indicated that contradictory press releases had adverse effects on the diagnostic outbreak response by creating confusion and that this should be avoided during the next outbreak. Most recommendations to improve a future outbreak response were made regarding the speed of the response. Seven laboratories suggested to create a system (e.g. a web portal at the networks’ websites) that allows access to relevant scientific data and information on and access to control reagents. Other suggestions included the development of an international concept, the establishment of a well-trained task-force, e.g. highly specialised treatment centres, and the quick identification of European experts and their contact information. Furthermore, it was noted that the transport to reference laboratories should be improved, and generic tests (e.g. pan-filovirus or syndrome-based, multiplex RT-PCR) should be advanced to improve identification of first cases without knowledge of the causative agent. In addition, one laboratory requested a formal recommendation from authorities in EU countries to downgrade the BSL of diagnostics for European travellers with unknown infections by one level. Finally, one laboratory observed that issues of trained staff were the result of inexperience and/or discomfort with BSL4 conditions and protocols owing to long periods between outbreaks. They proposed that access to (experimental) vaccines for the staff may alleviate some discomfort.

## Discussion

The retrospective assessment of the European laboratory response to the EBOV outbreak in West Africa is an important part of international processes to identify bottlenecks in the current operational procedures and to improve the laboratory response and its geographic coverage in future outbreaks [17,18]. Although six countries had no in-country or pre-agreed external access to EBOV diagnostics, this would not exclude these countries establishing an ad hoc collaboration in emergent situations. The fact that half of the laboratories had implemented EBOV diagnostics during the outbreak in West Africa illustrates considerable flexibility under urgent circumstances which was also supported by the availability of commercial diagnostic kits. However, a weakness identified was that the surplus resources needed for an adequate response were not matched with funding in half of the laboratories. Sustained national and EU funding mechanisms are needed to ensure adequate and robust laboratory preparedness, and rapidly deployable contingency funds are needed for outbreak response.

A wide range of molecular diagnostic tests was used by the 31 laboratories. Although the comparative quality of the tests could not be assessed here, in silico and laboratory comparative assessments of different tests for EBOV Zaire were conducted during and after the outbreak [11,16,19-21], with more information on test performances in clinical settings becoming available after the outbreak [22-25]. These provide points of action for improvement in European laboratories that have implemented suboptimal tests. The observation that only 14 laboratories confirmed all test outcomes is worrisome and needs improvement, especially in laboratories that had no previous or no extensive experience in EBOV diagnostics.

The level and type of accreditation of the EBOV diagnostic laboratories requires improvement as quality assurance of diagnostic procedures is an important aspect of such accreditation. In the EQA organised by WHO and ENIVD during the PHEIC, results of 28% of participant laboratories showed need for improvement [16], indicating that quality of the EBOV diagnostics is a concern and should be monitored and improved by e.g. training and additional EQAs in periods between outbreaks. Although multiple EQAs were organised by QUANDHIP before and during the outbreak, these were for BSL4 laboratories only, thereby overlooking the EBOV preparedness and response in the majority of European laboratories [9,26,27] (A. DiCaro, personal communication, June 2017). Finally, it should be kept in mind that future outbreaks with strains other than EBOV Zaire or with a divergent EBOV Zaire strain would require new assessments and provision of positive controls.

Biosafety concerns were indicated by the majority of the 25 laboratories without EBOV diagnostics as one of the reasons that made them refrain from diagnostic response to the outbreak. Ebola virus is listed as a RG4 pathogen, which requires high containment facilities for complex handling, while processing of diagnostic specimens can be done at BSL3 [28,29]. Especially when public health issues (e.g. rapid outbreak response) are at stake, daily practice increasingly follows a biorisk management approach, where the exact working conditions are based on boundaries set by local biosafety and biosecurity assessments and official audits, using the RG and BSL classifications as a basis [30,31]. In case BSL4 and BSL3 facilities are not available or other biosafety solutions must be implemented for practical reasons, early steps of a validated pathogen inactivation, as exemplified by one laboratory using bedside inactivation, should be considered and a biorisk assessment should result in protective measures which will be safe for workers and environments [32,33]. Indeed, 21 of 25 laboratories with EBOV diagnostics at BSL3 took extra precautionary measures. The questionnaire did, however, not allow to identify the motivation for these changes, e.g. whether it was done to increase the (perception of) biosafety or to acquire local permission to handle samples from biosafety staff. While laboratories in the EVD-LabNet and EMERGE JA networks with routine diagnostic workflows for a range of rare exotic viral diseases often have BSL3 laboratories, additional laboratory investigations required for patient management are typically not done in high containment facilities.

Collaboration among EU bodies and organisations and between laboratories is critical to ensure good outbreak response. This survey suggested a mostly positive view of collaboration in terms of sharing knowledge and materials, which indicates that the two EU laboratory preparedness and response networks provide added value. The most frequent recommendation to help improve preparedness in case of future outbreaks was to establish a central system that would allow quick and easy exchange of relevant, anonymised scientific data and control reagents. The availability of positive control materials was one of the main challenges EBOV diagnostic laboratories faced. It could be provided through establishment of such a portal on the networks’ member sites and accelerated access to materials via portals such as the European Virus Archive (EVAg) [34].

While the EBOV outbreak in West Africa was the largest on record, the risk for Europe was considered low [4,35,36]. Nevertheless, the outbreak activated a broad laboratory and clinical response in Europe. One approach to manage such response to an outbreak with limited risks could be to centralise such diagnostics in a limited number of BSL4 laboratories. In line with this, we observed that a vast majority of the samples tested for EBOV in the European laboratories were tested by the BSL4 laboratories. However, while samples from patients outside Europe were most likely to be sent to the BSL4 laboratories, local suspected cases were most likely to be tested in first-line, local diagnostic laboratories without BSL4 capacity but with the possibility for confirmatory testing in BSL4 laboratories. Furthermore, in the early phases of an outbreak, it is difficult to predict whether scaled up capacity will be needed. Also, many specialised diagnostic laboratories of both laboratory preparedness networks provide haemorrhagic fever differential diagnostics besides the BSL4 laboratories [8,10] and were called in to provide guidance for national and institutional level preparedness planning for EBOV patients.

## Conclusion

There are various national approaches for preparedness and an exchange of experiences could be useful. Therefore, the lessons learned from this survey and the indicated reasons for not performing EBOV diagnostics can be used to optimise the future response of clinical and specialised (containment) laboratories for high-threat pathogens.
